# Testing the assumptions of parametric linear models: the need for biological data mining in disciplines such as human genetics

**DOI:** 10.1186/s13040-019-0194-z

**Published:** 2019-02-11

**Authors:** Jason H. Moore, Trudy F. C. Mackay, Scott M. Williams

**Affiliations:** 1 Department of Biostatistics and Epidemiology, Institute for Biomedical Informatics, Perelman School of Medicine, University of Pennsylvania, Philadelphia, PA 19104-6021 USA; 2 Center for Human Genetics and Department of Genetics and Biochemistry, Clemson University, 114 Gregor Mendel Circle, Greenwood, SC 29646 USA; 3 Department of Epidemiology and Biostatistics, Case-Western Reserve University, 10900 Euclid Avenue, Cleveland, OH 44106 USA

All data science methods have specific assumptions that are made in order for their inferences to be valid. Some assumptions impact statistical significance testing and some influence the models themselves. For example, a fundamental assumption of linear regression is that the relationship between the independent and dependent variables is additive such that a unit increase in one leads to a unit increase in the other with some error that can be modeled using a normal distribution. The presence of a nonlinear relationship between the variables violates this assumption and can lead to inaccurate inferences. We demonstrate this here using a simple example from human genetics and then end with some thoughts about the role of biological data mining in revealing nonlinear relationships between variables.

One of the central questions of human genetics is the extent to which variation in a quantitative trait such as cholesterol levels or blood pressure is due to variations in the DNA sequence. Heritability is one measure that is used to assess the relative contributions of genetic and non-genetic (environmental) variation to trait variation. Heritability ranges from zero to one with a value of one indicating that all variation in the trait is attributable to genetic variation. In reality, we never see heritability this high because nearly all biological traits have one or more environmental components and are generally measured with some error. Heritability estimates that take into account all of the different types of genetic effects are referred to as broad-sense heritability (BSH). Additive genetic effects have historically received the most attention because they are useful for animal breeding and can be estimated from the correlation of the trait between pairs of relatives. An example of an additive genetic model would be a quantitative trait that has means of 10, 10.5, and 11 for genotypes *AA*, *AG*, and *GG*, respectively, at a single point in the DNA sequence. In this example, the *G* allele increases the mean by one half unit for each inherited copy. Twice the trait correlation among sibling pairs is an estimate of heritability due to additive genetic effects and is referred to as narrow-sense heritability (NSH). Twice the correlation is used here because siblings only share, on average, half their genes. Cousins share, on average, 12.5% of their genes and thus NSH is estimated from the trait correlation multiplied by eight.

**Fig. 1:**
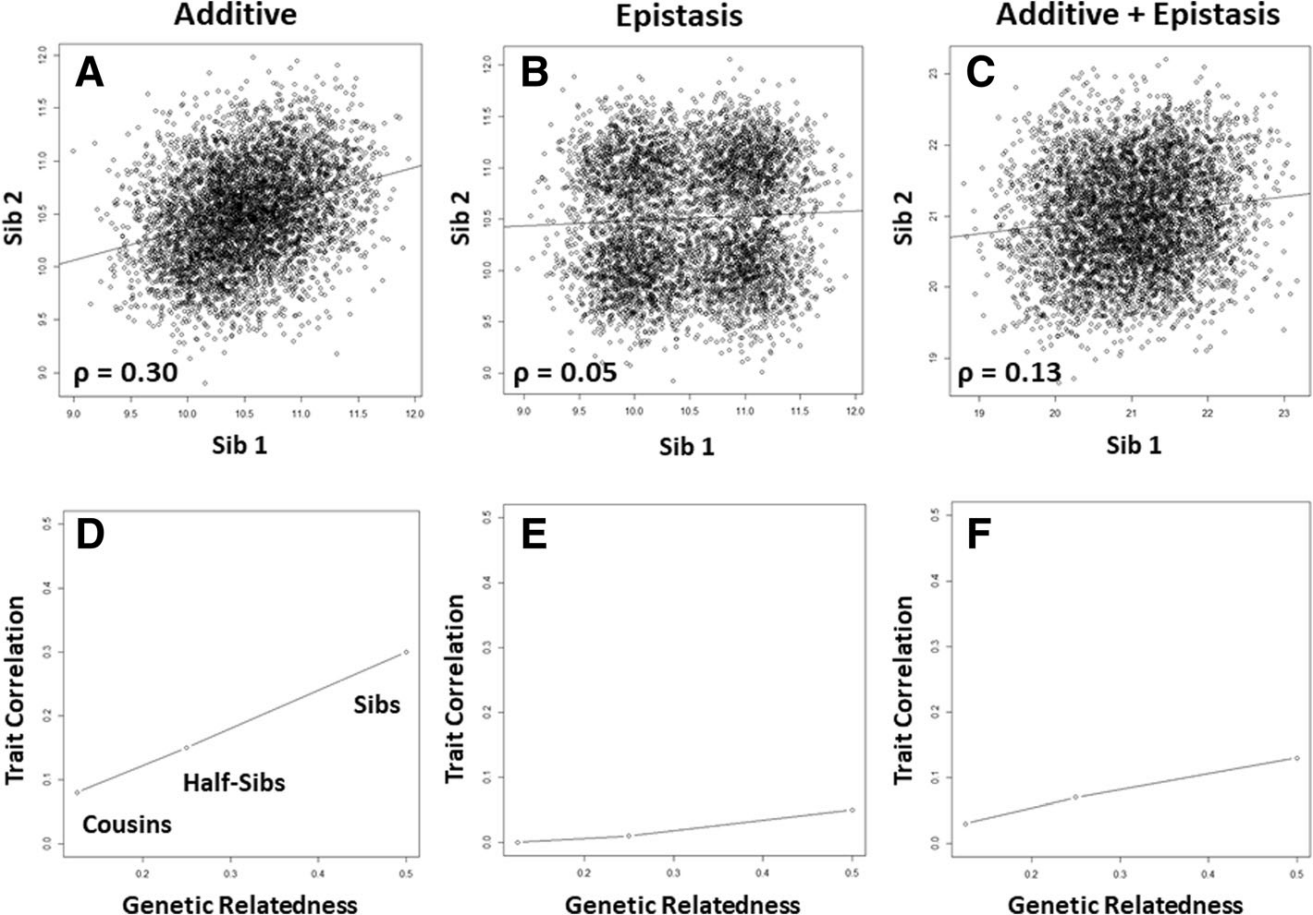
The top panels show the trait relationships between sib pairs simulated using additive (**a**), epistasis (**b**), and additive plus epistasis (**c**) models. Least squares fit regression lines and the correlation coefficient are shown for each. The bottom panels show the relationship between trait correlations estimated from sib, half-sib, and cousin pairs plotted against the expectation of their genetic relatedness. The panels **d**-**f** correspond to the genetic models labeled in **a**-**c**. Note that the linear trends in **c** and **f** are present despite more than 60% of the heritability being due to epistasis as a nonlinear genetic effect

To illustrate NSH we present the following simulation results using the genetic model presented above. Here, the genotypic means are 10, 10.5, and 11. We assume a total trait variance of 0.3. We assume the alleles (*A* and *G*) have equal frequencies of 0.5 in the population and assume genotype frequencies of 0.25 (*AA*), 0.5 (*AG*), and 0.25 (*GG*) that are consistent with Hardy-Weinberg expectations. We first simulated a single DNA sequence variation in 5000 unrelated parents using these genotype frequencies. We then simulated two children by drawing from the two alleles from each parent with 0.5 probability. We then simulated phenotypes for each parent and each sibling from a normal distribution with means and variance as described above for the additive genetic model. Figure 1a shows a scatterplot of the relationship of the trait between for each of the sibling pairs. Also shown is a least squares fit regression line with a slope and correlation of approximately 0.30. Using this correlation, we can estimate the NSH of this trait as 0.60. As validation, we can also use a linear model to estimate the variance component for the DNA sequence variation in the unrelated parents. Here, we coded genotypes *AA*, *AG*, and *GG* as 0, 1, and 2 so we can estimate the additive genetic variance component. The ratio of the additive variance over the total trait variance is an estimate of NSH. Here, this is equal to 0.60 which is exactly the heritability estimated from the trait correlation in the sib pairs. We confirmed this by estimating NSH from simulated half-sibs and cousins. We showed that the NSH for this additive genetic model drops in half and then in half again as the genetic relatedness of the relative pairs drops in half (Fig. 1d). The linear relationship between trait correlation and genetic relatedness of the relative pairs used for each estimate is considered by many to be a hallmark of an additive genetic model for the trait.

Any difference between BSH and NSH is due to other types of genetic effects such as deviation in the trait means due to dominance of an allele within a locus or epistasis (gene-gene interaction) between loci. Epistasis impacts trait variation through non-additive interactions between two or more DNA sequence variations. What is the impact of epistasis on relative pair correlations and estimates of NSH? To address this question, we carried out a similar simulation using an epistasis model such that the trait means are dependent on genotype combinations from two different DNA sequence variations. For the first genetic variant, we assume alleles (*A* and *G*) have equal frequencies of 0.5 and genotype frequencies of 0.25 (*AA*), 0.5 (*AG*), and 0.25 (*GG*). For the second genetic variant, we assume alleles (*T* and *C*) have equal frequencies of 0.5 and genotype frequencies of 0.25 (*TT*), 0.5 (*TC*), and 0.25 (*CC*). We further assume that these two genetic variants are on different chromosomes and thus unlinked, so the loci are not correlated (i.e., no linkage disequilibrium). We used an XOR (exclusive OR logic function) model for the epistasis effect such that the trait mean is 10 if the genotype at genetic variant one is *AG* OR the genotype at genetic variant two is *TC* but NOT both. Otherwise the trait mean is 11. This creates a pattern among the genotypic means such that both genetic variants are needed along with a nonlinear model to fully account for the genetic component of the trait variance. We assumed here the same trait variance of 0.3 and simulated parents and relative pairs as described above. Figure 1b shows a scatterplot of the relationship between the traits for each of the sibling pairs. Note the multimodal nature of the scatterplot that is due to having two different means for the genotype combinations. This is an extreme model for illustrative purposes and one may never see a pattern like this for a real biological trait given all the other genetic and environmental factors at play. This will be illustrated in the next model below. Also shown is a least squares fit regression line with a slope and correlation of approximately 0.05. Using this correlation, we can estimate the NSH of this trait as 0.10. Interestingly, the NSH estimates drop to zero in half-sibs and cousins (Fig. 1e). This is consistent with the variance components analysis in the parents that estimates the additive variance component to be zero suggesting that there is no additive component to the genetic variance as would be expected from this purely epistatic model. The slight correlation of 0.05 in the sibs is due to the slightly increased chance of them sharing the same genotypes at both genetic variants yielding the same genotypic means of 10 or 11. This creates slightly more subjects in the lower left quadrant and the upper right quadrant giving the least squares fit regression line a slightly positive slope (monozygotic twins would be all in the lower left and upper right quadrants). This slight linear pattern disappears completely in half-sibs and cousins. Coding the genotype combinations as 0 for those with a mean of 10 and 1 for those with a mean of 11 yields an epistatic variance component of 0.25. Since we know this is the generative model we can divide this by the sample trait variance of 0.34 yielding a BSH of 0.73. Thus, nearly 75% of the trait variation is due to the non-additive interaction between two DNA sequence variations. Very little if any of this heritability is accounted for in the estimate of the NSH. Thus, as expected, relative pair correlations tell us little about genetic effects due to this type of epistasis.

It is interesting to note that many biological traits exhibit a linear relationship in relative pairs that increases linearly with increasing genetic relatedness. What does this tell us about the nature of the genetic effects underlying the trait variation? Do observed linear relationships mean that the genetic effects are only additive at the exclusion of non-additive effects such as those coming from epistasis? To answer this question we combined the above simulations such that the trait from the additive simulation was added to the trait from the epistasis simulation yielding a new trait. We conducted the same heritability analysis as described above. Figure 1c shows a scatterplot of the relationship between the traits for each of the sibling pairs. Also shown is a least squares fit regression line with a slope and correlation of approximately 0.13 yielding a NSH estimate of 0.26. It is interesting to note that the scatterplot for this trait looks much more similar to the one shown in Fig. 1a. The only visible difference is that the correlation is not as strong. Thus, adding in the epistasis effect reduces the overall correlation but maintains the linear trend. The NSH estimate from half-sibs is 0.14 while cousins yield an estimate of 0.06. Thus, as with the purely additive genetic model, we see a linear increase in trait correlation and NSH with increasing genetic relatedness (Fig. 1f). The variance components analysis gives an additive component of 0.12 and an epistasis component of 0.24 for an overall genetic component of 0.36. Dividing the additive component by the overall variance of 0.55 gives a NSH of 0.22. Dividing the overall genetic component by the trait variance gives a BSH of 0.65. Thus, the epistasis component of the genetic model accounts for twice as much heritability as the additive.

The results of this last simulation illustrate that non-additive genetic effects can be hidden or buried in seemingly linear relationships, thus violating a fundamental assumption of parametric linear regression. This phenomenon has been previously observed [1]. Narrow-sense heritabilities on the order of 0.2 to 0.5 are commonly observed for a wide variety of human quantitative traits with estimates of BSH often higher suggesting non-additivity. For example, van Dongen et al. [2] estimated NSH for a number of metabolic traits in thousands of dizygotic twins that share half their genetic material and compared this to the BSH estimated from monozygotic twins that share all of their genetic material. The difference between these estimates is due to non-additive genetic effects such as dominance and epistasis. Several of the traits had fairly large differences. For example, triglycerides have an NSH of 0.33 and BSH of 0.59. Similarly, systolic blood pressure show NSH and BSH estimates of 0.37 and 0.60, respectively. These are remarkably similar to the estimates from our simulation. Similar differences between BSH and NSH for metabolic traits have been observed in mouse models [3]. There must be additive genetic effects contributing to the variability of these traits. However, epistasis and perhaps dominance effects cannot be ruled out as contributing to trait variation, as demonstrated here and has been pointed out previously [4]. This simple example highlights the importance of assumptions and the need for biological data mining methods from machine learning and artificial intelligence that make fewer assumptions about the nature of the models being constructed. Indeed, the use of data science methods such as machine learning and artificial intelligence is increasing as genetic studies of complex traits move from documenting the simple additive relationships to embracing the complexity of the genotype to phenotype mapping relationship that is likely to involve nonlinear genetic effects such as epistasis.
